# Ultrasound guidance for femoral venous access in electrophysiology procedures—systematic review and meta-analysis

**DOI:** 10.1007/s10840-019-00683-z

**Published:** 2019-12-10

**Authors:** Péter Kupó, Róbert Pap, László Sághy, Dalma Tényi, Alexandra Bálint, Dorottya Debreceni, Indranill Basu-Ray, András Komócsi

**Affiliations:** 1 Heart Institute, Medical School, University of Pécs, Ifjúság útja 13, Pécs, H-7624 Hungary; 2 Second Department of Internal Medicine and Cardiology Centre, Medical School, University of Szeged, Szeged, Hungary; 3 Department of Neurology, Medical School, University of Pécs, Pécs, Hungary; 4 St. Francis Hospital, Memphis, TN USA; 5 All India Institute of Medical Sciences, Virbhadra Marg, Rishikesh, Uttarakhand India

**Keywords:** Ultrasound-guided puncture, Electrophysiology procedures, Vascular access, Pulmonary vein isolation, Complications

## Abstract

**Purpose:**

The most common complications of electrophysiology (EP) procedures are related to vascular access. Our study aims to conduct a meta-analysis comparing ultrasound (US)-guided vs. palpation-based technique for femoral venous access in EP procedures.

**Methods:**

Electronic databases were searched and systematically reviewed for studies comparing femoral vein puncture with/without US in EP procedures. The primary outcome was the rate of major vascular complications; secondary outcomes were minor vascular complications, inadvertent artery puncture, postprocedural groin pain, and puncture time. Predefined subgroup analysis was conducted separately for patients undergoing pulmonary vein isolation procedure (PVI). A random-effects model was used to derive risk ratios (RR) with 95% confidence interval (CI).

**Results:**

Nine studies involving 8232 patients met our inclusion criteria. Compared with the standard technique, the use of US reduced major vascular complications (from 2.01 to 0.71%, *p* < 0.0001). The rate of minor vascular complications (RR = 0.30, 95% CI, 0.14–0.62, *p* = 0.001) and inadvertent artery puncture were lower with US-guided puncture (RR = 0.31, 95% CI, 0.17–0.58, *p* = 0.0003). Puncture time was shorter (mean difference = − 92.1 s, 95% CI, − 142.12 – − 42.07 s, *p* = 0.0003) and postprocedural groin pain was less frequent (RR = 0.57, 95% CI, 0.41–0.79, *p* = 0.0008) in the US group. Subgroup analysis of patients undergoing PVI also showed significant reduction of major vascular complications (RR = 0.27, 95% CI, 0.12–0.64, *p* = 0.003) and inadvertent artery puncture (RR = 0.35, 95% CI, 0.21–0.59, *p* < 0.0001).

**Conclusion:**

Real-time US-guidance of femoral vein puncture in EP procedures is beneficial: it reduces major and minor vascular complications, inadvertent artery puncture, postprocedural groin pain, and puncture time.

## Background

The most common complications of electrophysiology (EP) procedures are related to the vascular access. These may interfere with the quality of life of the patients and often prolong hospitalization [1, 2]. The rate of vascular access-related complications varies depending on the definition and the type of the procedure. Atrial fibrillation (AF) ablation carries higher risk of vascular access complications compared with other EP procedures [1]. This may be explained by the combination of uninterrupted oral anticoagulation and intraprocedural systemic anticoagulation to reduce the risk of thromboembolic complications, in addition to multiple large-bore access sites for the procedure compared with most EP procedures. This exposes patients undergoing AF ablation to a higher risk of bleeding complications, ranging from 1 to 13% depending on the cohort [1, 3–6].

**Fig. 1:**
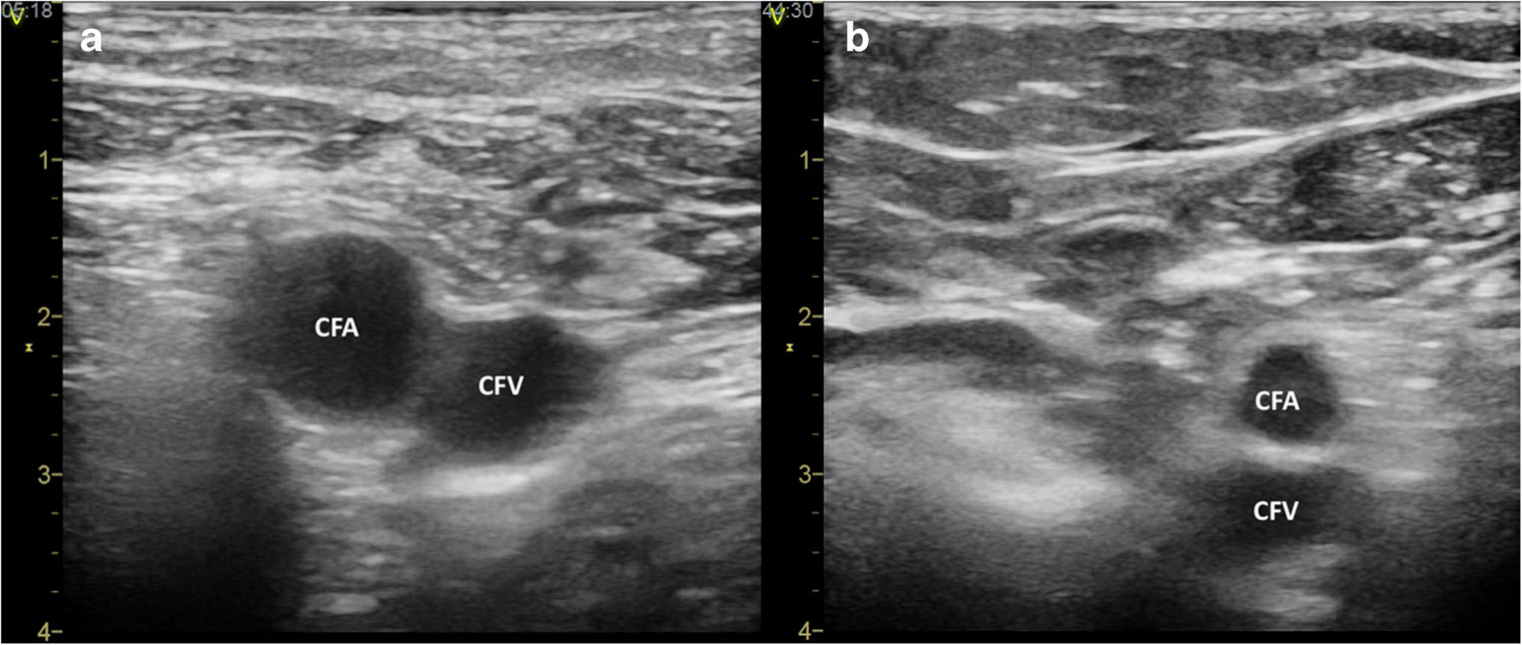
Illustrative examples of two-dimensional ultrasound images of the femoral vessels with expected (Panel **a**) and unexpected (Panel **b**) localization. CFA common femoral artery, CFV common femoral vein

Supplementing the traditional anatomy landmark-based vascular puncture, an ultrasound (US) guidance offers potential benefits including prevention of vascular access-related complications. Using an US-gel-filled sterile sleeve covered vascular probe connected to a portable ultrasonograph allows direct and real-time visualization of the inguinal region. US may clarify the anatomy of the femoral vessels and the surrounding structures and identify variations that may interfere with the success of the puncture (Fig. 1). In addition, placing the probe perpendicular over the inguinal region and the femoral vein, it is possible to follow the needle during the puncture to guide and correct its course [7]. US-guided femoral puncture has a short learning curve and does not interfere with the normal workflow of EP procedures [7]. Earlier studies reported a lower rate of inadvertent arterial puncture, a higher rate of first-pass success, and a decreased risk of complications associated with the use of US [8–10].

A previous meta-analysis of observational trials showed a 60% and 66% reduction in major and minor vascular complications using US guidance for femoral vein access in EP procedures [11]. Since this study, further important data—including a randomized controlled trial—have been published comparing US-guided vs. standard technique [12].

Therefore, in the present study, we aimed to extend the earlier analyses in order to better characterize the impact of US-guidance at cannulation of the femoral vein during EP procedures.

## Methods

### Search strategy

Electronic databases (Medline, Excerpta Medica Database (EMBASE), Cochrane Register of Controlled Trials (CENTRAL), and Scopus) were searched for relevant articles published between January 2000 and April 2019 using a search strategy that combined text word and MeSH heading. The search string was “vascular” and “ultrasound” or “ultrasonography” and “electrophysiology or electrophysiological” or “catheter ablation.” No language restriction was used. Furthermore, we extended the search with the reference lists of the relevant studies and reviews, editorials, letters, and also relevant abstracts. We performed the analyses according to the PRISMA guideline [13].

### Data abstraction and statistical analysis

Both randomized controlled and observational trials—regardless of their prospective/retrospective design—comparing real-time US-guided to conventional, anatomical landmark and palpation-guided technique for femoral vein puncture in EP procedures were identified. Selection and data abstraction were done independently by two reviewers (P.K. and A.K.). Disagreements were resolved by consensus.

The meta-analysis was conducted by using Review Manager (RevMan) Version 5.3 software (Cochrane Collaboration, London, UK). A random-effects model was used to derive risk ratios (RR) with 95% confidence interval (CI) on dichotomous outcomes and mean difference on continuous data. Heterogeneity was tested with a chi square heterogeneity statistic for which a *p* value < 0.2 was considered potentially heterogeneous. Consistency was assessed by the I^2^ statistic, which describes the percentage of total variation across studies that is due to heterogeneity rather than due to chance. Inconsistency was described as low, moderate, and high, based on I^2^ values of 25, 50, and 75%, respectively.

The primary outcome was the presence of major vascular complications (groin hematoma, arteriovenous fistula, and pseudoaneurysm). Hematoma was considered to be a major vascular complication if it met type 2 or higher Bleeding Academic Research Consortium (BARC) criteria (requiring nonsurgical, medical intervention by a health care professional; leading to hospitalization or increased level of care, or prompting evaluation) [14]. Secondary outcomes were minor vascular complications (groin hematomas < BARC 2), inadvertent artery puncture, groin pain, and puncture time. Predefined subgroup analysis was conducted separately for patients undergoing pulmonary vein isolation (PVI) procedure. The review protocol was registered in the PROSPERO database under the registration number of CRD42019139143.

## Results

**Fig. 2:**
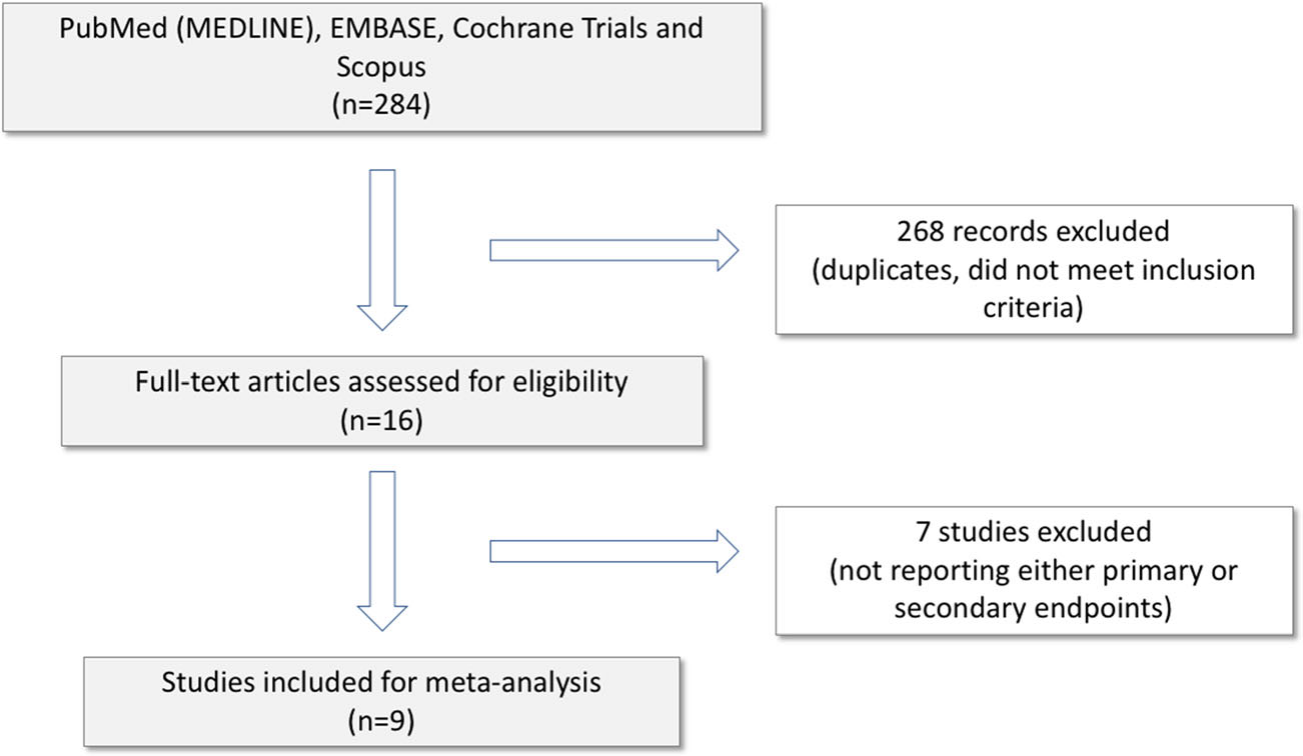
Flowchart of study selection

Nine studies involving 8232 patients met our inclusion criteria, published between 2013 and 2018. One study was randomized and eight were observational, non-randomized (Table 1) [12, 15–22]. The results of the literature search are summarized in Fig. 2.

**Table 1:** Study and patients’ characteristics of the included trials

First author	Year	Design	Procedure number	Procedure type	Operators	Operators’ experience with US-guided femoral vein puncture	US system	Puncture needle	Mean age	Male (%)	Mean BMI	AC thera-py (%)	Peri-procedural AC regimen	AP the-rapy (%)
Tanaka-Esposito	2013	Single center, retrospective	3420	PVI	EP fellows	NA	NA	18-gauge	NA	77.6	NA	NA	UI/I	32
Errah-Mouni	2014	Single center	300	EPS/Abl	2 Experts, 2 EP fellows	NA	NA	18-gauge	64.6 ± 17	65.3	28.2 ± 4.5	64	UI	3
Wynn	2014	Single center, prospective	309	PVI	NA	2-week period during live cases	SonoSite S-ICUTM, Fujifilm SonoSite Inc., WA, USA	NA	58.9 ± 10.2	72.5	29.6 ± 4.6	100	UI/I	NA
Rodriguez-Munoz	2015	Single center, prospective	36	EPS/Abl	1 EP fellow, 3 Residents in cardiology	3 Procedures	Acuson Freestyle, Siemens Medical Solutions USA, Inc., CA, USA	NA	69.3 ± 19.4	69.4	26.0 ± 4.6	52.7	NA	NA
Sharma	2016	Single center, prospective	720	EPS/Abl	NA	NA	SonoSite S-ICUTM, Fujifilm SonoSite Inc., WA, USA	NA	58 ± 16	53.0	30.0 ± 7.0	59	UI	32
Dussault	2016	Conference abstract	1196	PVI/CTI abl	EP fellows	NA	NA	MicroUltz, micropuncture needle (manufacturer NA)	61.2 ± 10.7	71.5	NA	100	UI	NA
Yamagata	2017	Multicenter, randomized	320	PVI	5 Experts, 6 EP fellows	20–50 Procedures	Vivid I, GE Health Medical, Horten, Norway	18-gauge	63.0 ± 8.7	61.4	29.6 ± 5.2	100	UI	NA
Ströker	2018	Single center	1435	PVI	Experts, EP fellows	NA	NA	NA	60.0 ± 12.0	65.1	27.0 ± 4.0	56	UI	19
Mohanty	2018	Conference abstract	496	PVI	NA	NA	NA	NA	NA	0	NA	100	UI	NA

*Abl*, ablation; *AC*, anticoagulant; *AP*, antiplatelet; *BMI*, body mass index; *CTI*, cavotricuspid isthmus; *EP*, electrophysiology; *EPS*, electrophysiology study; *I*, interrupted; *NA*, not available; *PVI*, pulmonary vein isolation; *UI*, uninterrupted; *US*, ultrasound

**Fig. 3:**
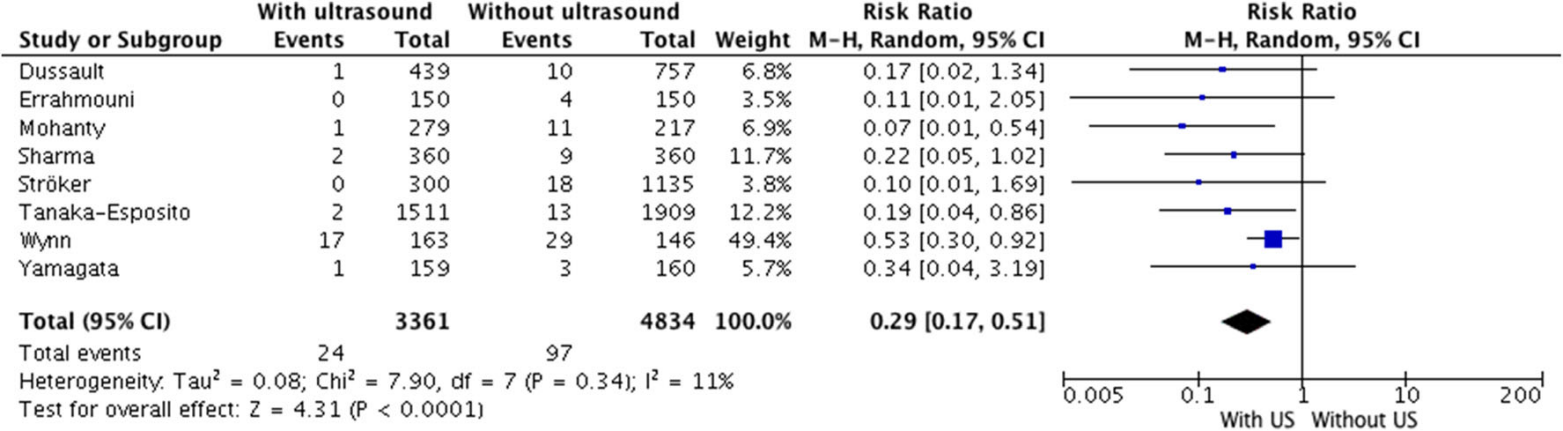
Major vascular complications. US ultrasound

Compared with the standard technique, the use of US for femoral vein puncture significantly reduced the rate of major vascular complications from 2.01 to 0.71%. Calculated from this, 71% of relative risk reduction (RR = 0.39, 95% CI, 0.17–0.51, *p* < 0.0001, Fig. 3), the number needed to treat (NNT) was approximately 77; that is, the US-guided puncture has to be applied in 77 patients to avoid 1 major adverse event.

**Fig. 4:**
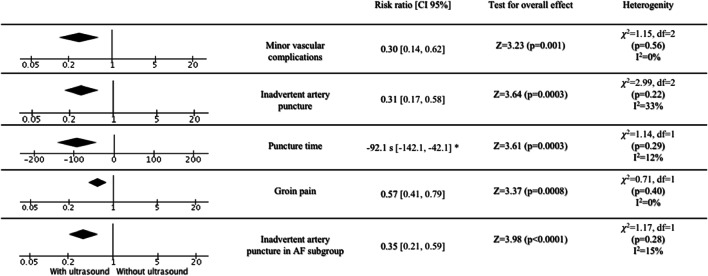
Summary of outcomes of secondary endpoints. AF atrial fibrillation. *Mean difference (95% CI)

Risk of minor vascular complications with US guidance showed a reduction from 1.49 to 0.45% (RR = 0.30, 95% CI, 0.14–0.62, *p* = 0.001, Fig. 4). The risk of inadvertent arterial puncture was reduced from 19.7 to 5.93% (RR = 0.31, 95% CI, 0.17–0.58, *p* = 0.0003) compared with the standard group (Fig. 4). The time required for the puncture was shorter (mean difference = − 92.1 s, 95% CI, − 142.12 – − 42.07 s, *p* = 0.0003, Fig. 4) and groin pain was less frequent (22.22 vs. 13.04%, RR = 0.41, 95% CI, 0.41–0.79, *p* = 0.0008) in the US group (Fig. 4).

**Fig. 5:**
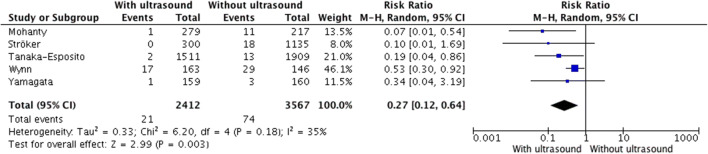
Major vascular complications in the subgroup of patients undergoing pulmonary vein isolation

Subgroup analysis of patients undergoing PVI also showed significant reduction of major vascular complications (2.07 vs. 0.87%, RR = 0.27, 95% CI, 0.12–0.64, *p* = 0.003, Fig. 5) and inadvertent artery puncture (19.28 vs. 6.52%, RR = 0.29, 95% CI, 0.17–0.50, *p* = 0.00001, Fig. 4). Among patients undergoing catheter ablation for AF, the NNT was 83. The evaluation of secondary outcomes in the subgroup analysis could not be performed due to the lack of data.

Based on the results of Chi^2^ and I^2^ tests, the sample proved to be homogenous and consistent, except for data regarding inadvertent arterial puncture, where moderate inconsistency could be detected. Funnel plot analyses showed no sign of possible publication bias.

## Discussion

Our meta-analysis demonstrated that the use of US guidance for vascular access among patients undergoing EP procedures reduces the relative risk of major vascular complications by 71% compared with the standard, anatomical landmark-guided technique. In addition, US-guided femoral vein puncture significantly reduced puncture time and improved outcomes regarding minor vascular complications, inadvertent arterial puncture, and postprocedural groin pain. In the subgroup analysis of patients undergoing PVI, the rates of major vascular complications and inadvertent artery puncture were significantly lower in the US-guided group.

Procedures involving puncture of major vessels are increasingly used in various fields of medicine. This is a tendency that besides opening possible new treatment options, it also exposes patients to a certain risk for complications. Data from the American Society of Anesthesiologists Closed Claims Project database indicates that the majority of central venous catheter placement-associated complications are vascular injuries [23]. In a previous review, the overall complication rate reached 15% for central venous catheterization [24]. This high frequency is partially related to the increasing rate of interventions requiring anticoagulant and antiplatelet medications, as well as to the limitations of the conventional palpation-based puncture technique.

The anatomical orientation points-guided puncture is considered to be the standard during femoral venous access, although examining bony landmarks and femoral arterial pulsation may not be sufficient for the localization of the femoral vein in a considerable proportion of cases. An analysis of the inguinal region computed tomographic scans showed that the femoral artery overlaps the femoral vein in two-thirds of the patients [25]. These individual variations may lead to unsuccessful puncture or various complications if direct visualization is not performed.

The US-guided approach allows direct visualization of femoral vessels with a higher rate of successful venous puncture. An analysis of four studies comparing the US-guided to the anatomical landmarks-guided technique for femoral vein cannulation showed that the use of US is associated with improved success rate, however, in these reports there were no significant differences in the complication rates, puncture time, arterial punctures, or hematomas [10]. Guidelines for the Prevention of Intravascular Catheter-Related Infections—developed by a task force comprising members from the Society of Critical Care Medicine, the Society for Healthcare Epidemiology of America, and the American Society of Critical Care Anesthesiologists—recommended the use of US-guidance during central venous catheter insertions [26].

Electrophysiology procedures may represent a field where the safety aspects of the central venous access site are of paramount interest. The most common major complications of EP procedures are related to vascular access. These may require intervention and result in prolonged hospitalization [1, 27, 28]. The prevalence of these complications varies depending on the type of procedure. Vascular access-related complications have been described in 0.3–0.4% of supraventricular tachycardia ablations [1, 29], and 0.4–4.7% of premature ventricular complex/ventricular tachycardia ablations [1, 30, 31]. Patients undergoing AF ablation procedures experience a vascular access complication rate of 1–13% depending on the definition [4, 5, 32, 33].

Catheter ablation for AF not only has the most commonly performed ablation procedure but also has specific considerations regarding the vascular access complications [27, 34].

Besides the large-bore catheters and multiple access sites used for these procedures, in order to prevent ischemic complications, the recommended treatment comprises uninterrupted oral anticoagulant administration [35]. Oral and procedural anticoagulation may augment the importance and interfere with the treatment of injuries suffered during the access and result in more frequent bleeding complications [27].

Improving the security of the catheter ablation remains a primary goal in electrophysiology. With a potential for easy adaptation in the EP laboratory environment, vascular US offers a quick and inexpensive technique with a steep learning curve. It does not increase the duration of the procedure, moreover—as our results showed—puncture time and thus the skin-to-skin measured procedure time can be reduced. For a detailed description of technical details, we refer to the review article of Wiles et al. describing the technique of US-guided femoral vein puncture with special regards to its application in the EP laboratory [7, 36]. Despite the obvious benefits, the routine use of US for femoral vein access has not been widely adopted among the electrophysiologists.

In light of the fact that the number of EP procedures is increasing, prevention of vascular access-related complications is desirable not only to improve safety outcomes but also to reduce additional costs of the management of complications [37]. We found that the risk reduction achievable with the US-assisted approach is reasonably high and the NNT to prevent major vascular complications is relatively low, supporting its routine use during femoral vein puncture for vascular access in EP procedures.

Our findings demonstrating benefits of the US guidance for EP procedures are in line with the results of a previous meta-analysis. Sobolev et al. performed an analysis of 4 trials including a total of 4605 patients comparing US-guided femoral vein cannulation in EP procedures to the anatomical landmark-based technique. The US-guided femoral vein puncture was associated with a 60% reduction of major vascular bleeding and a 66% reduction of minor vascular complications [11]. Since the publication of that analysis, several observational studies were published that were allowed to characterize the effect estimates with higher statistical power. Moreover, we could include data from the only randomized, controlled trial (RCT) in this field. In the multicenter, prospective ultrasound-guided femoral vein accessibility, safety and time (ULTRA-FAST) trial, 320 patients undergoing catheter ablation for AF were randomized to US-guided vs. conventional femoral vein puncture [12]. The use of US was associated with preferable intraprocedural outcomes; however, there was no difference in the major complication rates presumably due to the lower-than-expected complication rate in the conventional arm.

The present meta-analysis has some limitations to be acknowledged. Firstly, only one randomized study was included and the majority of data originate from observational studies. This may introduce potential biases and/or effects of unmeasured confounders. In general, observational studies are more precise but supposed to be more subject to bias. Importantly, we found very similar outcomes in the RCT compared with the observational studies. The lack of heterogeneity in this aspect supports the fact that the treatment effects of the US-guided approach are similar, independent from trial design, and not affected by potential bias.

Important differences also may exist in patient demographics that might affect outcomes and are not accounted for in this analysis. Use of a random-effects model can help mitigate the potential effect of heterogeneity and the high level of significance supports the validity of the results. Secondly, secondary outcomes were not reported by all of the included studies limiting further analysis of potential mechanisms. Finally, data regarding the operators performing the puncture and especially data on operators’ previous experience with US-guided technique were also insufficient despite the probable impact of a learning curve effect.

## Conclusion

In conclusion, our meta-analysis including 8232 patients demonstrated that real-time US-guidance of femoral vein puncture in EP procedures is beneficial by reducing major and minor vascular complications, inadvertent artery puncture, postprocedural groin pain, and puncture time. These data may substantiate recommendations for the routine use of US in EP procedures.
